# What You Don’t Know Can Hurt You: Uncertainty Impairs Executive Function

**DOI:** 10.3389/fpsyg.2020.576001

**Published:** 2020-10-06

**Authors:** Jessica L. Alquist, Roy F. Baumeister, Dianne M. Tice, Tammy J. Core

**Affiliations:** ^1^Psychological Sciences, Texas Tech University, Lubbock, TX, United States; ^2^Department of Psychology, The University of Queensland, Brisbane, QLD, Australia; ^3^Department of Psychology, Brigham Young University, Provo, UT, United States

**Keywords:** uncertainty, executive function, self-regulation, self-control, ego depletion

## Abstract

Three studies demonstrated that situational uncertainty impairs executive function on subsequent unrelated tasks. Participants were randomly assigned to either uncertain situations (not knowing whether they would have to give a speech later, Studies 1-2; uncertain about how to complete a task, Study 3) or control conditions. Uncertainty caused poor performance on tasks requiring executive function that were unrelated to the uncertainty manipulation. Uncertainty impaired performance even more than certainty of negative outcomes (might vs. definitely will have to make a speech). A meta-analysis of the experimental studies in this package found that the effect is small and reliable. One potential explanation for this effect of uncertainty on executive function is that uncertainty is a cue for conserving effort.

## Introduction

Uncertainty is a common experience for decision-makers in many contexts, including health care (e.g., Babrow et al., 1998; Han et al., 2011), business (Erdem and Keane, 1996; Bloom et al., 2018), military conflict (Posen, 2016), environmental protection (Ascough et al., 2008; Polasky et al., 2011), government economic policy (Stockhammer and Grafl, 2010), real estate (Thanh et al., 2018), and sports (Knobloch-Westerwick et al., 2009). Elucidating the effects of uncertainty could therefore have practical value as well as build scientific theory. If uncertainty itself causes cognitive fatigue, that could impair effortful decision-making — quite possibly in ways of which the decision maker would be unaware. The present research is designed to test the hypothesis that uncertainty impairs executive function.

### Executive Function and Ego Depletion

Executive functions are the top-down processes required to change or override automatic responses (Diamond, 2013). Executive function is required for processes such as decision-making, self-control, and initiative (Baumeister, 2002). The hypothesis that executive function can be impaired because of low energy, akin to the folk notion of willpower, was proposed in the 1990s (e.g., Baumeister et al., 1998; Muraven et al., 1998). The state of impaired performance was dubbed ego depletion. Over the past two decades, hundreds of studies were published showing various kinds of ego depletion effects (for reviews, see Hagger et al., 2010; Baumeister and Vohs, 2016). Recently, there has been a lively debate about the existence, effect size, and mechanism of the ego depletion effect (Beedie and Lane, 2012; Kurzban et al., 2013; Inzlicht et al., 2014, 2015; Xiao et al., 2014; Carter et al., 2015; Inzlicht and Berkman, 2015; Cunningham and Baumeister, 2016; Dang, 2016, 2017; Hagger et al., 2016; Lurquin et al., 2016; Dang et al., 2017, 2020; Garrison et al., 2019). Ego depletion can be replicated in many contexts and laboratories but also does not occur invariably, and so extending the theory to include moderating factors and parallel processes is a high priority.

Multiple studies have extended ego depletion to decision-making. Vohs et al. (2014) showed that making effortful decisions led to subsequent impairments in executive function. Pocheptsova et al. (2009) showed, conversely, that ego depletion stemming from effortful tasks impaired subsequent decision making, effectively shifting people toward low-effort responses to decision dilemmas. They found that depleted participants maximized on a single dimension rather than integratively compromising to maximize across multiple dimensions, they postponed decisions, and they failed to think carefully so as to prevent logically irrelevant information from biasing their choices. Making decisions impairs executive function, and previous acts of executive function impair decision-making.

The initial theorizing about ego depletion assumed that executive function was impaired because the person’s energy had been expended and was too low to permit further exertions. That view is no longer considered tenable. A variety of moderators have been shown to reduce or eliminate the effects of depletion. If participants performed poorly on a second executive function task because they were truly unable to control themselves, factors such as motivation on the second task (Muraven and Slessareva, 2003; Park et al., 2008; Vohs et al., 2012), self-affirmation (Schmeichel and Vohs, 2009), and positive affect induced between tasks (Tice et al., 2007) would be unlikely to eliminate ego depletion effects (Inzlicht and Schmeichel, 2012). Participants’ likelihood of failing at self-control is also increased by believing self-control is limited (Job et al., 2010; but cf. Vohs et al., 2012) and by believing one has expended energy on a previous task (Clarkson et al., 2010). Beliefs about the nature of self-control or the task one has completed would be unlikely to moderate the effect of a first task on a second task if the resource was “used up” and unavailable for further exertion.

Some recent theories of ego depletion suggest that the effect of a first task on a second is due to cost-benefit calculations, or a combination of such calculations and limited resources (Beedie and Lane, 2012; Kurzban et al., 2013; Inzlicht et al., 2014; Shenhav et al., 2017; André et al., 2019). There is evidence that ego depletion effects observed in the lab are due to conservation, not a thoroughgoing exhaustion of a resource. The self may have expended some energy and though it is far from being entirely out of fuel it seeks to conserve what remains. Previous research on conservation has shown that when participants have already expended effort and anticipate additional tasks requiring self-control, they perform worse on the current task and better on the anticipated task than participants who are surprised with an additional task (Muraven et al., 2006). One reason people fail at sequential executive function tasks may be that once they have expended some effort, they begin to conserve their remaining energy for future tasks that may have high priority. In this way, mental energy is similar to physical energy, in which muscles feel tired and athletes begin conserving their energy long before their muscles even approach true exhaustion (Evans et al., 2015).

### Uncertainty

There are hundreds of published laboratory studies on ego depletion, but most of them have induced the state by requiring participants first to engage in a task requiring self-control. We sought to broaden the potential focus of this area by showing that encountering uncertainty can cause impairments in executive function similar to those caused by a task specifically designed to be effortful. Determining other ways of producing depletion-like effects may provide additional information about the mechanism of the effect (Milyavskaya et al., 2019).

Uncertainty involves an individual lacking important information (Bar-Anan et al., 2009). One may lack information about whether, when, or where something will happen, or what will happen. One may have multiple pieces of conflicting information and lack information about which is true or should be weighted most heavily. One may know the details of the situation but be uncertain about how best to respond effectively.

Uncertainty may cue conservation via The Behavioral Inhibition System. The Behavioral Inhibition System is a motivational system that becomes activated in response to situations that are conflicted or uncertain and pauses progress on uncertain or conflicted goals (Gray and McNaughton, 2000; Corr et al., 2013; Hirsh and Kang, 2016). Halting progress when circumstances are uncertain can protect an organism from encountering harm (trying to get a piece of food a predator is guarding), and it can preserve energy for the yet-undetermined demands of the situation. Previous research has shown that thinking about an issue about which one was uncertain impairs task performance through activation of the Behavioral Inhibition System (Alquist et al., 2018).

A recent review of animal research by Anselme and Güntürkün (2018) showed that in environments marked by uncertainty about food, animals shifted toward conservation strategies, including caching and hoarding food, eating more, and gaining weight. Thus, uncertainty causes conservation of energy resources even in quite simple animals. Such animals are presumably unable to engage in complex projections of multiple possible futures (or, indeed, cost-benefit calculations amid multiple alternatives). Indeed, a recent experiment showed that even humankind’s closest and presumably highly intelligent ape relatives were unable to learn to understand the future as containing multiple alternative possibilities — unlike human children, who quickly grasped the multiplicity of alternatives (Redshaw and Suddendorf, 2016). Responding to uncertainty therefore does not require complex understanding, and the impulse to conserve resources in response to uncertainty may be unconscious and automatic.

Energy conservation would likely be an adaptive response to uncertainty. Presumably people (like other animals) evolved to conserve energy because one could not be sure of always having enough resources. Inadequate energy exposed one to multiple risks, including impaired immune function and death. The more uncertain the future, the more adaptive it would be to conserve energy generally so as to be able to cope with unknown developments. If ego depletion typically occurs because the human body is reluctant to expend energy that it might need later on, then uncertainty should heighten this tendency because it heightens the possibility of future demands. By definition, uncertainty means not knowing what to expect — and so it is impossible to know how much energy will be required. Therefore, the adaptive response to uncertainty would be to conserve.

### Present Research

Our studies manipulated initial exposure to uncertainty and then measured self-regulatory performance. In Studies 1 and 2, we randomly assigned participants to be either certain or uncertain about what they would be doing later in the study and measured executive function using a skill-based game, namely Operation (Study 1), and solvable anagrams (Study 2). In Study 3, we randomly assigned participants to be uncertain or certain about how to respond to task prompts by making the instructions mismatched to the response situation, and then measured their subsequent executive function by measuring persistence on unsolvable puzzles. We also report a meta-analysis testing whether the effect is reliable across all measures. These manipulations are a departure from much of the previous research on depletion, because we are not directly manipulating how effortful the initial task is.

Social psychology has recently shifted toward new methodological criteria, including pre-registration of methods and hypotheses, and larger samples. This research was conducted prior to those changes, back when the best practices emphasized convergence across multiple methods in different studies. Publication was delayed because we sought to establish the mediating process based on the initial theory that uncertainty would evoke extra mental work and emotion regulation to account for multiple alternative possibilities. We were unable to find evidence of that mechanism. A complete list of the mediators and moderators tested in these studies is presented in the Supplementary Materials. The revised theory, that uncertainty serves as a cue to stimulate conservation, has emerged as a more plausible alternative, particularly in light of the recent review by Anselme and Güntürkün (2018).

## Study 1: Operating Under Uncertainty

Study 1 experimentally manipulated uncertainty in order to test whether uncertainty impaired executive function. The type of uncertainty being tested in this study involved uncertainty in which the participant was waiting for important information. Specifically, participants in the uncertain condition were left uncertain about whether they would have to give a speech later in the study (Core et al., 2018). Participants in the certain conditions were either told that they would soon have to give a speech or were told that they would not have to give a speech.

Expecting to give a speech is a highly aversive and stressful circumstance for many people. Previous research has shown that participants assigned to anticipate and then give a speech had an increased heart rate and cortisol as compared to baseline (Kirschbaum et al., 1993). Expecting a speech has been shown to affect participant’s performance on tasks requiring executive function. Participants assigned to anticipate giving a speech learn more slowly on the Iowa Gambling task (Preston et al., 2007) and score lower on decision-making tasks (Starcke et al., 2008). A simple prediction would be that the aversiveness of the experience, and therefore the degree of impairment, would be felt in direct proportion to the anticipated likelihood of the aversive (speech) outcome. Thus, definitely having to give a speech would be the worst, definitely not having to speak would be the best, and uncertainty would fall in between. In order to show that uncertainty *per se* was depleting, we predicted that uncertainty would be at least as detrimental to subsequent executive function as the certain expectation of having to speak.

Executive function was measured using the board game Operation, which has been used in previous studies (e.g., DeWall et al., 2008; Englert and Bertrams, 2013). The game requires participants to remove pieces from a board as quickly and with as few errors as possible. Inhibition is required for participants to stay focused on the task and carefully avoid making errors. Balancing the need to finish things quickly and the desire to do them well is relevant in everything from meeting deadlines at work to performing non-board-game surgery to getting a manuscript submitted to a scientific journal. We predicted that participants who were uncertain about whether they would be giving a speech would make more errors and take more time to complete the task than participants in the no speech condition — and would also be equal to or worse than participants in the definite speech condition.

### Method

#### Participants

Fifty participants (22 women; 28 men) participated in this study in exchange for course credit. Four participants were excluded from the final sample: two participants who reported knowing there were no other participants in the experiment; one participant who came into the lab very sick; and one participant who arrived too late to complete the study. The final sample had an average age of 19.87 (*SD* = 4.87). 10.9% identified as Latino or Hispanic Latino. Participants’ races were 4.3% Asian,13.6% Black or African American, 78.3% white, 2.2% more than one race and 2.2% unknown or not reported.

#### Procedure

##### Uncertainty manipulation

All participants were told that some participants would be giving speeches while other participants rated those speeches (Core et al., 2018). They were told that they would be completing the communication task later in the study, but to save time, they would be assigned their condition now. In the speech and no speech conditions, participants were told, “You are participant number ___, and it says here that you are in the speech (no speech) condition. Let’s start on the intelligence task, and when you’re done, we’ll move to another room for the communication task.” In the uncertain condition, the experimenter acted flustered and said, “Hmm. You are supposed to be participant number ___, but I don’t see your number on here anywhere. I have a master sheet in the other room with all the numbers on it. I’m going to start you on the next task, and I’ll go get the sheet while you are working.” This left the participants in the uncertain condition uncertain about whether they would be giving a speech later in the study.

##### Executive function

###### Operation

Executive function was measured using the board game Operation (DeWall et al., 2008). Participants were told they were doing the Operation task as a measure of hand-eye coordination. Each participant was asked to try removing one piece for practice before the task began. Participants were asked to remove all the pieces from the board as quickly as possible. The experimenter recorded the time the participant spent working and the number of times the participant sounded the buzzer by hitting the sides of a piece’s space on the game board. For consistency across all studies, we report each measure (e.g., time and errors; number attempted and solved) separately rather than computing composite scores (DeWall et al., 2008, 2011).

##### Competence

We worried that participants may have withheld effort in the uncertain condition because they viewed the experimenter as incompetent (given that the experimenter did not know the condition). In order to test this possibility, participants were asked to respond to the question, “How competent was the researcher who administered your study today?” on a scale of 1 (not at all) to 5 (extremely). Responses were made on the computer to reduce participants’ concern that the experimenter would see their responses.

##### Additional measures

In addition to the measures reported here, exploratory mediators and moderators that were included in this and the following studies are reported in the Supplementary Materials.

### Data Analyses

We conducted ANOVAs comparing the means in the three conditions on errors, time, and perceived experimenter competence. We also conducted planned comparisons between individual conditions.

### Results

#### Operation Performance

As predicted, ANOVA revealed a significant difference among conditions on the number of errors participants made (i.e., the number of times they sounded the buzzer), *F*(2, 43) = 4.14, *p* = 0.02, *η^2^* = 0.16, 90% CI[0.01, 0.30], See Table 1. Planned comparisons revealed that participants in the uncertain condition (*M* = 21.92, *SD* = 5.84) made significantly more errors than participants in the no speech condition (*M* = 15.92, *SD* = 7.50), *t*(43) = 2.15, *p* = 0.04, *d* = 0.86, 95% CI[0.05, 1.66]. Planned comparisons also indicated that participants in the uncertain condition made significantly more errors than participants in the speech condition (*M* = 15.10, *SD* = 7.29), *t*(43) = 2.77, *p* = 0.01, *d* = 0.98, 95% CI[0.25, 1.69].

**TABLE 1:** Study 1: Means and standard deviations across uncertainty conditions on errors and time during the operation task.

	Uncertain	Speech	No Speech
	
	*M (SD)*	*M (SD)*	*M (SD)*
Number of errors	21.92_*a*_ (5.84)	15.10b (7.29)	15.92_*b*_ (7.50)
Time in seconds	229.31 (37.51)	214.08 (75.05)	214.71 (61.20)

*Means with different subscripts are significantly different at p < 0.05.*

There was no significant difference among conditions on the amount of time participants took to complete the Operation task, *F*(2, 43) = 0.29, *p* = 0.75, *η^2^* = 0.01, 90% CI[0.00, 0.08]. There was also no significant relationship between the time participants took and the number of errors they made, *r* = 0.09, *p* = 0.56, 95% CI[-0.21, 0.37]. Thus, it appears that only the error measure was sensitive to the manipulation, and how long it took participants to finish it was relatively unaffected by it.

#### Competence

There were no differences among conditions in participants’ perceptions of the experimenter’s competence, *F*(2, 43) = 0.86, *p* = 0.43, *η^2^* = 0.04, 95% CI[0.00, 0.16]. There was also no correlation between perceived experimenter competence and the time participants spent or the number of errors they made on the Operation task, all *p*’s > 0.54.

### Discussion

Participants who were uncertain about whether they would have to give a speech made significantly more errors on the Operation task than both participants who knew they would not have to give a speech and participants who knew they would have to give a speech. There were no differences between conditions in time spent on the task. We acknowledge that multiple values resulting from the dependent variable increase the risk of Type 1 error. In order to address this concern, we include all values (for example, in this study, number of errors and time spent) for dependent variables in the meta-analysis of studies on page 17.

Participants who were uncertain about whether they would have to give a speech showed poorer executive function than participants who knew for sure that they would have to give a speech. This suggests that, as far as executive function is concerned, it is actually better to be sure of a negative outcome than to know a negative outcome is possible.

A possible alternative explanation for the predicted results would be that participants in the uncertain condition inferred that the experimenter was incompetent, based on the experimenter in that condition not knowing what treatment had been assigned to them. This alternative was not supported by the ratings of the experimenter competence, which showed no difference by condition.

## Study 2: Uncertainty and Solvable Anagrams

Study 1 found evidence that waiting to find out if one was giving a speech impaired executive function more than knowing one would have to give a speech. Study 2 was designed to extend this effect to another measure of executive function, as a conceptual replication.

Participants’ executive function was measured using a series of solvable anagrams. Working through a daunting task under the pressure of a deadline demands that people use executive function to avoid distractions and to focus their attention on the task at hand. Solving anagrams also requires working memory because it involves trying letter combinations in different orders. Maintaining focus and persevering despite failure also requires inhibition for successful anagram performance. Anagram attempts have been used as a measure of executive function in previous research (Muraven et al., 1998), and we specifically used solvable anagrams because we wanted to include a measure for which success was possible.

### Method

#### Participants

Ninety-two participants (70 women; 22 men) participated in this study in exchange for course credit. Three participants were excluded from the final sample because they reported knowing that they would not have to give a speech. The final sample was 22.4% Hispanic or Latino. Participants’ race representation was: 1% American Indian/Alaska Native, 2% Asian, 3.1% Black, 69.4% White, 6% More than one race, 18.5% Unknown or not reported. Participants’ mean age was 18.35 (*SD* = 0.80).

#### Measures and Procedure

##### Uncertainty manipulation

Uncertainty was manipulated using the same procedure as in Study 1.

##### Executive function

In order to measure participants’ executive function, participants were given a set of fifty solvable five-letter anagrams and were asked to solve as many as possible in ten minutes. There was a blank line next to each anagram where participants were asked to put their solution. Any line on which the participants wrote an attempted solution was coded as an attempted anagram, and each anagram solved correctly was considered a completed anagram.

##### Self-reported uncertainty

After working for ten minutes on the anagrams, participants were asked to answer some questions before they began the speech task. Participants were asked to respond to the statement, “Earlier in the study, I was uncertain about whether or not I‘d be giving a speech” on a scale of 1 (strongly disagree) to 9 (strongly agree).

#### Data Analyses

We ran ANOVAs comparing the means in the three conditions on anagrams attempted, anagrams solved, and self-reported uncertainty. We also ran planned comparisons between individual conditions.

### Results

#### Anagram Performance

**FIGURE 1:**
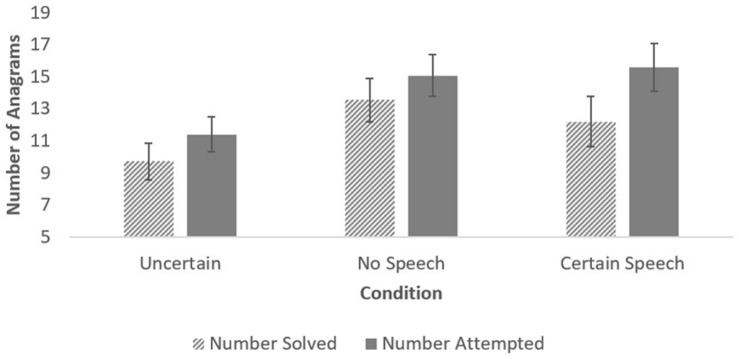
Anagrams solved and attempted by condition in study 2. Error bars show standard errors.

ANOVA revealed a significant effect of condition on the number of anagrams participants attempted, *F*(2, 86) = 3.59, *p* = 0.03, *η^2^* = 0.08, 90% CI[0.004, 0.17], See Figure 1. Participants in the uncertain condition (*M* = 11.37, *SD* = 5.38) attempted significantly fewer anagrams than participants in the no speech condition (*M* = 15.07, *SD* = 8.21), *t*(86) = 2.18, *p* = 0.03, *d* = 0.54, 95% CI[0.05, 1.03]. Participants in the uncertain condition also attempted significantly fewer anagrams than participants in the speech condition (*M* = 15.57, *SD* = 7.48), *t*(86) = 2.26, *p* = 0.03, *d* = 0.61, 95% CI[0.07, 1.14]. This replicates the finding from Study 1 that executive function suffered more when participants knew a bad outcome was possible than when they knew the same outcome was definite.

The effect of condition on the number of anagrams solved was not significant, *F*(2, 86) = 2.49, *p* = 0.09, *η^2^* = 0.05, 90% CI[0.00, 0.13]. Planned comparisons showed that participants in the uncertain condition (*M* = 9.70, *SD* = 5.52) solved significantly fewer anagrams than participants in the no speech condition (*M* = 13.54, *SD* = 7.99), *t*(86) = 2.17, *p* = 0.03, *d* = 0.53, 95% CI[0.04, 1.02]. Although participants in the uncertain condition solved fewer anagrams than those in the definite speech condition, (*M* = 12.19, *SD* = 8.69), the results were not significant, *t*(86) = 1.29, *p* = 0.20, *d* = 0.35, 95% CI[-0.18, 0.88]. The difference between the speech and no speech conditions was also not significant, *t*(86) = 0.89, *p* = 0.38, *d* = 0.26, 95% CI[-0.31, 0.82].

Thus, participants who were uncertain about whether they would have to give a speech attempted fewer anagrams than participants in the speech and no speech conditions and solved fewer anagrams than participants in the no speech condition.

#### Self-Reported Uncertainty

ANOVA revealed a significant degree of variation among conditions in how uncertain participants reported feeling about their role in the communication task, *F*(2, 86) = 4.26, *p* = 0.02, *η^2^* = 0.09, 90% CI[0.01.18]. Planned contrasts revealed that participants in the uncertain condition (*M* = 7.17, *SD* = 2.01) reported feeling more uncertain than participants in the no speech condition (*M* = 5.61, *SD* = 2.64), *t*(86) = 2.87, *p* = 0.005, *d* = 0.77, 95% CI[0.23, 1.31]. Although the means were in the predicted direction, participants in the uncertain condition did not report feeling significantly more uncertain than participants in the speech condition (*M* = 6.81, *SD* = 1.94), *t*(86) = 0.61 *p* = 0.54, *d* = 0.15, 95% CI[-0.33, 0.63].

### Discussion

Participants who were uncertain about whether they would have to give a speech made significantly fewer attempts to solve the anagrams than both participants who knew they would not have to give a speech and participants who knew they would have to give a speech. Thus, once again, uncertainty in the form of possibly bad news produced worse performance than definite bad news. Uncertain participants also solved fewer anagrams than those in the no speech condition and those in the definite speech condition, though the last difference was not significant. These results provide evidence that uncertainty about what one will be required to do impairs people’s performance on subsequent measures of executive function.

Participants in the uncertain condition reported being significantly more uncertain than participants in the no speech condition. Those in the definite speech condition reported levels of uncertainty intermediate between the two (and not significantly different from either). Participants in the speech condition may have reported uncertainty about their potential performance on the speech task rather than uncertainty about which task they would be completing.

## Study 3: Response Uncertainty

Study 3 manipulated uncertainty by giving participants a task where it was either clear how they should respond (control condition) or unclear how they should respond (uncertain condition). Participants were briefly shown a colored square on the computer. They were then asked to complete the math problem associated with the color they just saw. For example, participants saw a yellow square for one second and then the instructions on the computer read “Please complete the equation associated with the color you just saw: Blue: 2 × 6; Green: 12 × 3; Yellow: 10 × 7; Red: 9 × 9.” For participants in the control condition, all twenty trials showed colored squares that fit clearly into the four categories provided (blue, green, yellow and red). However, for participants in the uncertain condition, twelve of the twenty trials included colors that did not fit the colors provided (e.g., orange, blue-green, purple). We predicted that participants who performed the unclear task would feel significantly more uncertain than participants who were given the clear task — and this uncertainty would carry over to cause impairments in performance on a subsequent, unrelated task.

Executive function was measured using persistence on puzzles that (unbeknownst to participants) were unsolvable. Persistence on a difficult (in this case, impossible) task requires inhibition because individuals have to override the impulse to quit (Baumeister et al., 1998). Executive function includes the effortful overriding of one’s responses, particularly with the goal of changing them according to some standard. Persistence requires overriding any desire to quit in order to force oneself to keep striving despite discouragement and failure. Because the tasks were unsolvable, discouragement and failure would continue unabated as long as the person persisted. We predicted that participants who were given the unclear version of the task would subsequently spend significantly less time persisting on the puzzles and make fewer attempts to solve the puzzle than participants who were given the clear version of the task. We also predicted that this relationship would be mediated by participants’ self-reported uncertainty from the first task.

### Method

#### Participants

Fifty-one participants (15 men, 36 women) participated in this study in exchange for course credit. One participant was excluded from analyses for recognizing that the puzzle was unsolvable. The final sample had an average age of 18.65 (SD = 1.41).

#### Procedure

##### Uncertainty manipulation

The uncertainty manipulation was programed using MediaLab research software (Jarvis, 2006). The experimenter told participants that the purpose of the study was to understand how people reason through different kinds of puzzles. Participants were shown a square of color on the computer screen for one second. They were then shown the names of four colors next to four math problems and were asked to complete the math problem associated with the color of the square they had seen previously. Participants in the control condition saw colors that clearly matched the colors listed for all twenty trials. Participants in the uncertain condition were shown colors that did not clearly fit the colors listed (e.g., blue-green) for twelve of the twenty trials.

To prevent participants from stopping to ask about the ambiguous colors, participants were told that they would be timed and should work as quickly as possible. To increase participants’ motivation to do well, all participants were told they would earn twenty-five cents each time they answered correctly, and they could earn up to five dollars on the task. At the end of the study, all participants were given $5.

##### Executive function

###### Persistence

After completing the colored square task, participants were given an unsolvable tracing puzzle as a measure of executive function (Baumeister et al., 1998). In order to convince participants that the puzzle was solvable, the experimenter completed a solvable tracing puzzle in front of the participant as an example. Participants were given the instructions from Baumeister et al. (1998), and were told that if they wished to stop before they finished, they should ring the bell on the table. Participants were provided with a stack of paper containing many copies of the same unsolvable puzzle and a highlighter. The experimenter left the room, and began timing the amount of time the participant persisted before ringing the bell. Any participant still working after 30 minutes was interrupted and asked to continue with the rest of the study (3 participants worked until the limit: 1 in the uncertain condition, 2 in the control condition). Each copy of the puzzle that was marked with the highlighter was coded as one attempt to solve the puzzle.

##### Manipulation check

After the unsolvable puzzle, participants were asked to respond to the question, “When you were completing the task with the colored squares and the math problems, how uncertain did you feel?” on a scale of 1 (not at all uncertain) to 5 (very uncertain). Last, participants were probed for suspicion, debriefed about the purpose of the study, paid, and dismissed.

#### Data Analysis

We ran t-tests comparing the uncertain and control conditions on puzzle attempts, time, and self-reported uncertainty. We also tested whether the effects of condition on attempts and time were mediated by self-reported uncertainty.

### Results

#### Executive Function

##### Persistence

There was a significant difference between the uncertain and control conditions on the number of attempts made at solving the unsolvable tracing puzzle, *t*(48) = 2.28, *p* = 0.03, *d* = 0.63, 90% CI[1.05, 16.86], See Table 2. As predicted, participants who had been given the ambiguous task subsequently made fewer attempts (*M* = 18.00, *SD* = 11.41) than participants in the control condition (*M* = 26.95, *SD* = 16.38). A parallel effect was found for the measure of time spent working on the puzzles, but it was not significant, *t*(48) = -1.73, *p* = 0.09, *d* = 0.49, 95% CI[-8.46,0.63]. Participants in the uncertain condition (*M* = 10.61 minutes, *SD* = 7.35) spent less time on the unsolvable tracing puzzle than participants in the control condition (*M* = 14.53 minutes, *SD* = 8.63).

**TABLE 2:** Study 3: Means and standard deviations across conditions on attempts and time on unsolvable puzzles and on self-reported uncertainty.

	Uncertain	Control
	
	*M (SD)*	*M (SD)*
Attempts	18.00* (11.41)	26.95* (16.38)
Time on puzzles	10.61 (7.35)	14.53 (8.63)
Self-reported uncertainty	2.68* (0.86)	1.82* (0.91)

*p < 0.05.

#### Manipulation Check

There was a significant difference between the uncertain and control conditions in how uncertain participants felt about the first task, *t*(48) = 3.42, *p* = 0.001, *d* = 0.97, 95% CI[0.36, 1.37]. Participants in the ambiguous color condition (*M* = 2.68, *SD* = 0.86) reported being significantly more uncertain about the task than participants in the control condition (*M* = 1.82, *SD* = 0.91). Thus, the manipulation had the intended effect.

#### Mediation

Self-reported uncertainty was negatively correlated with both the number of attempts, *r* = -0.28, *p* = 0.05, and the amount of time people spent on the unsolvable puzzle, *r* = -0.42, *p* < 0.01. We tested the mediating effect of condition on the dependent variables through self-reported uncertainty using the method recommended by Preacher and Hayes (2004). The indirect effect of condition on time persisting was estimated to be 164.67, 95% CI [58.72, 403.53]. Because the confidence interval does not include zero, this suggests that the indirect effect of condition on time persisting through self-reported uncertainty was significant. The indirect effect of condition on number of puzzle attempts through self-reported uncertainty was estimated at 2.19, 95% CI [-1.55, 6.65]. Because the confidence interval contains zero, this indicates that the indirect effect of condition through self-reported uncertainty on puzzle attempts was not significant.

The amount of uncertainty participants felt about the task mediated the relationship between their assigned condition and how long they persisted on the subsequent unsolvable puzzles, but not on how many attempts they made to solve the puzzle. This suggests that condition decreased time persisting by increasing uncertainty.

### Discussion

Study 3’s results converged with those of the first two studies, despite changes in both manipulation and dependent measure. We found once again that uncertainty impaired subsequent executive function. Participants who performed one task hampered by unclear, ambiguous instructions later quit more quickly on a separate, unrelated task, as compared to people for whom the initial instructions could be clearly and easily followed.

It is possible that other differences between the uncertain and certain condition (such as task difficulty) could have contributed to the poorer performance on the second task. However, the effect of condition on executive function was mediated by how uncertain participants reported feeling, which suggests that uncertainty is at least part of the reason for impaired performance on the subsequent task. The manipulation check indicated that the manipulation increased uncertainty, though not to extreme levels (2.68 out of maximum 5). This suggests that even a moderate amount of uncertainty is enough to impair executive function.

## Meta-Analysis

We conducted a meta-analysis to test the effect of uncertainty on executive function across studies. When two outcomes were measured (e.g., time persisting and number of attempts), both were included in the analyses for significance, and the effect sizes were combined following the guidelines for combining dependent effects (Rosenthal and Rubin, 1986). For studies with multiple contrasts, the comparison between the uncertain and speech conditions (the more conservative test) was used. We found that the effect of uncertainty on executive function was reliable, *Z* = 4.67, *p* < 0.001, and the effect size was small, *r* = 0.101.

## General Discussion

Three studies provided evidence that uncertainty impaired performance on subsequent self-control tasks, even though those tasks had no logical relationship to the previous experience of uncertainty. Participants who were left uncertain about whether they would have to give a speech showed impaired performance on the game Operation (Study 1) and on an anagram completion task (Study 2), as compared to participants in no speech or speech control conditions. In Study 3, participants who were given an unclear task gave up faster on a second unrelated task than participants who were given a clear first task.

The effect of uncertainty on self-control was robust across different experiences of uncertainty. We tested manipulations of uncertainty involving an unclear task and uncertainty in the form of waiting to find out whether one will have to perform an anxiety-producing task. The convergence across these different experiences of uncertainty increases confidence in the general conclusion that being uncertain leads to impairments in self-regulatory performance, even in domains unrelated to the uncertainty. We also found that feelings of uncertainty mediated the effects of uncertainty manipulations on subsequent self-control (Study 3).

Studies 1 and 2 indicated that the effects of uncertainty go beyond merely raising the possibility of a bad outcome. They showed that the uncertain possibility of a bad outcome caused more impairment than certainty that the bad outcome would occur. Specifically, participants who thought they might have to make a speech performed significantly worse than participants who faced the worst possible outcome, namely a definite assignment that they would have to give a speech. Uncertainty in the form of anticipating the *possibility* of a negative outcome thus impaired self-control more severely than certain anticipation of the same negative outcome. Previous research has found that people are willing to pay *less* for a chance at one of two outcomes (e.g., you will receive a $50 or $100 gift certificate) than for the worse outcome guaranteed (e.g., you will receive a $50 gift certificate; Gneezy et al., 2006; Simonsohn, 2009). Although choosing a less-positive certain outcome over an uncertain outcome may seem irrational, it may sometimes be worth avoiding the psychological costs of experiencing uncertainty, namely impairments to executive function.

### Alternative Explanations

Our findings cannot establish whether uncertainty actually causes cognitive fatigue or merely mimics it. In practice, the difference to the decision-maker may be trivial. In either case, the person may automatically shift toward less effortful modes of deciding (Pocheptsova et al., 2009; Pohl et al., 2013). These conserve energy but reduce the role of rational input into the decision process.

One might argue that our results were obtained because uncertainty distracted participants in the moment, rather than necessarily impairing self-control on a subsequent task. Study 3 provides some evidence against this. Although in studies 1 and 2, participants were uncertain while completing the dependent measure of self-control, in Study 3, the uncertainty manipulation and subsequent measure of self-control were distinct tasks. Participants first completed the task on which they were made to feel uncertain. Self-regulatory deficits were found on a subsequent, separate, and unrelated task, and it is unlikely that while participants were working to solve the figure tracing puzzles they were still ruminating about whether the color squares they had seen earlier had been red or blue. Also, as noted above, Study 1’s measures also spoke against the alternative interpretation that the uncertainty condition caused people to think the experimenter was incompetent.

### Implications

The idea that uncertainty can impair self-control has diverse potential for advancing ego-depletion theory. We assume that participants in our studies did not deliberately, knowingly lower their performance based on exposure to unrelated uncertainty. Unconscious processes presumably mediated the link between experiencing uncertainty in one context and seeking to conserve volitional resources in another. Hence decisions about whether to exert effort may be influenced by multiple factors, only some of which are conscious.

The analogy of executive function fatigue to a muscle was creatively extended by Evans et al. (2015). They noted that feelings of muscular tiredness are only loosely linked to the physical condition of the muscle. Some brain processes presumably keep track of exertion and create a feeling signal of tiredness to promote energy conservation. Our findings fit well with the suggestion that ego depletion also may be only distantly related to the actual availability of energy resources. Instead, various cues associated with past and future demands may prompt the individual to curtail self-regulatory effort. Our findings suggest that uncertainty may be one such cue. To be sure, conserving resources may often be a highly adaptive response to uncertainty — even though in our experimental situation, it brought no benefits.

We cited evidence that people in uncertain conditions suffer problems of mental and physical health (e.g., Wiggins et al., 1992; Burgard et al., 2009). Impaired self-control may prove to be a mediating factor, if people struggling with uncertainty cease to control their eating and alcohol consumption, curtail their health behaviors, mistreat relationship partners, or fail to regulate their emotions. The present research suggests that delivering clear news quickly to patients (when possible) may make it easier for them to make important choices or follow demanding treatment regimens than if information is delayed or unclear.

The importance of self-control to smooth societal functioning suggests that large-scale uncertainty could have a variety of troublesome effects. Uncertainty may disproportionately be present for certain social classes (such as low-income groups) or may periodically affect society as a whole (such as in times of economic downturns, political turmoil, or public health crises). Crime, addiction, intimate partner violence, and general impulsivity might all increase. These would compound the problems facing society that gave rise to the original uncertainty.

### Limitations and Future Directions

There are a few reasons to interpret the presented results with caution. As mentioned in the introduction, these studies were conducted before new standards for pre-registration and large sample sizes were established. Research conducted in the future on this topic should follow the current standards in the field. It is also worth noting that the internal meta-analysis is only based on a small number of studies. Although the hypothesis was always that uncertainty would impair performance, we tested a number of exploratory hypotheses across studies about potential mediators and moderators. None of these predominantly non-significant results advanced the theory, and all are reported in the Supplementary Materials. We also note that the effect of uncertainty was replicated, so the impairment of executive function caused by uncertainty is robust, even though we were unable to find evidence of a specific mechanism in these studies.

Although uncertainty was our primary independent variable, we cannot claim to have studied all forms of uncertainty. Undoubtedly there are some differences among the varieties of uncertainty (Kahneman and Tversky, 1982), even in our studies. However, for both manipulations, participants were made aware that they lacked highly relevant information. It is this lack of knowledge that ultimately results in poorer subsequent self-control, regardless of the exact kind of knowledge that is lacking. We deliberately broadened our investigation to encompass multiple forms of uncertainty (rather than operationalizing it in the same way in all studies) to increase generalizability and ensure that our results were not due to one particular method or one kind of uncertainty. Research using other measures of uncertainty (e.g., a scratched vs. unscratched lottery ticket) have shown that people are more likely to choose “wants” over “shoulds” when uncertain, which provides additional evidence that uncertainty has a negative effect on self-control (Milkman, 2012). The convergence of results across these different uncertainties increases our confidence that the pattern is indeed a relatively general one.

Future work may test whether there are situations in which some kinds of uncertainty would not impair self-control. For example, uncertainty about a definite positive outcome (e.g., uncertainty about which online interaction partner said which positive thing about the participant) has been shown to increase the duration of positive affect (Wilson et al., 2005; Kurtz et al., 2007). Because positive mood has been shown to eliminate the effects of one act requiring self-control on subsequent self-control (Tice et al., 2007), it is possible that the net effect of a purely positive uncertainty may be neutral or even restorative. However, when a negative possibility exists, the present evidence suggests that people will be less likely to perform well at executive function if they have recently been or are currently uncertain.

## Conclusion

Uncertainty increases the difficulty of decision-making (Shafir, 1994). When all relevant facts are known, decision processes can be a fairly straightforward product of logic, preference, and goals or values. Often, however, decisions must be made when key facts are lacking (Orasanu and Connolly, 1993). Uncertainty hampers the decision maker directly, because it makes it difficult to calculate which option will yield best results. Our findings suggest a second way in which uncertainty impairs decision makers: It makes them act as if they had cognitive fatigue.

## Data Availability Statement

The raw data supporting the conclusions of this article will be made available by the authors, without undue reservation.

## Ethics Statement

The studies involving human participants were reviewed and approved by Florida State University Institutional Review Board; Texas Tech Review Board. The patients/participants provided their written informed consent to participate in this study.

## Author Contributions

DT, JA, and RB discussed and developed the idea. JA designed Studies 1 and 3 with input from RB and DT and designed Study 2 with input from all other co-authors. DT designed the manipulation from Study 3. JA and RB wrote the introduction and discussion, with input from the other co-authors. JA executed Studies 1–3, and analyzed and wrote the methods and results for Studies 1 and 3. TC analyzed and wrote the methods and results for Study 2.

## Conflict of Interest

The authors declare that the research was conducted in the absence of any commercial or financial relationships that could be construed as a potential conflict of interest.

## References

[B1] Alquist J. L. (2010). *What You Don’t Know Can Hurt You: Uncertainty Depletes Self-Control Resources.* Master’s thesis, Florida State University, Tallahassee, FL.

[B2] Alquist J. L. Baumeister R. F. McGregor I. Core T. J. Benjamin I. Tice D. M. (2018). Personal conflict impairs performance on an unrelated self-control task: lingering costs of uncertainty and conflict. *J. Exp. Soc. Psychol.* 74 157–160. 10.1016/j.jesp.2017.09.010 29662250 PMC5898441

[B3] André N. Audiffren M. Baumeister R. F. (2019). An integrative model of effortful control. *Front. Syst. Neurosci.* 13:79. 10.3389/fnsys.2019.00079 31920573 PMC6933500

[B4] Anselme P. Güntürkün O. (2018). How foraging works: uncertainty magnifies food-seeking motivation. *Behav. Brain Sci.* 42 1–106. 10.1017/S0140525X18000948 29514723

[B5] Ascough J. C. Maier H. R. Ravalico J. K. Strudley M. W. (2008). Future research challenges for incorporation of uncertainty in environmental and ecological decision-making. *Ecol. Model.* 219 383–399. 10.1016/j.ecolmodel.2008.07.015

[B6] Babrow A. Kasch C. R. Ford L. A. (1998). The many meanings of *uncertainty* in illness: toward a systematic accounting. *Health Commun.* 10 1–23. 10.1207/s15327027hc1001_1 16370987

[B7] Bar-Anan Y. Wilson T. D. Gilbert T. D. (2009). The feeling of uncertainty intensifies affective reactions. *Emotion* 9 123–127. 10.1037/a0014607 19186925

[B8] Baumeister R. F. (2002). Ego depletion and self-control failure: an energy model of the self’s executive function. *Self Identity* 1 129–136. 10.1080/152988602317319302

[B9] Baumeister R. F. Bratslavsky E. Muraven M. Tice D. M. (1998). Ego depletion: is the active self a limited resource? *J. Pers. Soc. Psychol.* 74 1252–1265. 10.1037/0022-3514.74.5.1252 9599441

[B10] Baumeister R. F. Vohs K. D. (2016). Strength model of self-regulation as limited resource: assessment, controversies, update. *Adv. Exp. Soc. Psychol.* 54 67–127. 10.1016/bs.aesp.2016.04.001

[B11] Beedie C. J. Lane A. M. (2012). The role of glucose in self-control: another look at the evidence and an alternative conceptualization. *Pers. Soc. Psychol. Rev.* 16 143–153. 10.1177/1088868311419817 21896791

[B12] Bloom N. Floetotto M. Jaimovich N. Saporta-Eksten I. Terry S. J. (2018). Really uncertain business cycles. *Econometrica* 86 1031–1065. 10.3982/ecta10927 30516143

[B13] Burgard S. A. Brand J. E. House J. S. (2009). Perceived job insecurity and worker health in the United States. *Soc. Sci. Med.* 69 777–785. 10.1016/j.socscimed.2009.06.029 19596166 PMC2757283

[B14] Carter E. C. Kofler L. M. Forster D. E. McCullough M. E. (2015). A series of meta-analytic tests of the depletion effect: self-control does not seem to rely on a limited resource. *J. Exp. Psychol. Gen.* 144 796–815. 10.1037/xge0000083 26076043

[B15] Clarkson J. J. Hirt E. R. Jia L. Alexander M. B. (2010). When perception is more than reality: the effects of perceived versus actual resource depletion on self-regulatory behavior. *J. Pers. Soc. Psychol.* 98 29–46. 10.1037/a0017539 20053029

[B16] Core T. J. Price M. M. Alquist J. L. Baumeister R. F. Tice D. M. (2018). Life is uncertain, eat dessert first: uncertainty causes uncontrolled and unemotional eaters to consume more sweets. *Appetite* 131 68–72. 10.1016/j.appet.2018.09.006 30195822

[B17] Corr P. J. DeYoung C. G. McNaughton N. (2013). Motivation and personality: a neuropsychological perspective. *Soc. Pers. Psychol. Compass* 7 158–175. 10.1111/spc3.12016

[B18] Cunningham M. R. Baumeister R. F. (2016). How to make nothing out of something: analyses of the impact of study sampling and statistical interpretation in misleading meta-analytic conclusions. *Front. Psychol.* 7:1639. 10.3389/fpsyg.2016.01639 27826272 PMC5079083

[B19] Dang J. (2016). Commentary: a multilab preregistered replication of the ego-depletion effect. *Front. Psychol.* 7:1155. 10.3389/fpsyg.2016.01155 27535004 PMC4971805

[B20] Dang J. (2017). An updated meta-analysis of the ego depletion effect. *Psychol. Res.* 82 645–651. 10.1007/s00426-017-0862-x 28391367 PMC6013521

[B21] Dang J. Barker P. Baumert A. Bentvelzen M. Berkman E. Zinkernagel A. (2020). A multilab replication of the ego depletion effect. Social psychological and personality science. *Adv. Online Pub.* (in press). 10.1177/1948550619887702 PMC8186735 34113424

[B22] Dang J. Liu Y. Liu X. Mao L. (2017). The ego could be depleted, providing initial exertion is depleting: a preregistered experiment of the ego depletion effect. *Soc. Psychol.* 48 242–245. 10.1027/1864-9335/a000308

[B23] DeWall C. N. Baumeister R. F. Mead N. L. Vohs K. D. (2011). How leaders self-regulate their task performance: evidence that power promotes diligence, depletion, and disdain. *J. Pers. Soc. Psychol.* 100 47–65. 10.1037/a0020932 20919772

[B24] DeWall C. N. Baumeister R. F. Vohs K. D. (2008). Satiated with belongingness? Effects of acceptance, rejection, and task framing on self-regulatory performance. *J. Pers. Soc. Psychol.* 95 1367–1382. 10.1037/a0012632 19025289 PMC2597411

[B25] Diamond A. (2013). Executive functions. *Annu. Rev. Psychol.* 64 135–168. 23020641 10.1146/annurev-psych-113011-143750 PMC4084861

[B26] Englert C. Bertrams A. (2013). Too exhausted for operation? Anxiety, depleted self-control strength, and perceptual-motor performance. *Self Identity* 12 650–662. 10.1080/15298868.2012.718865

[B27] Erdem T. Keane M. P. (1996). Decision-making under uncertainty: capturing dynamic brand choice processes in turbulent consumer goods markets. *Mark. Sci.* 15 1–20. 10.1287/mksc.15.1.1 19642375

[B28] Evans D. R. Boggero I. A. Segerstrom S. C. (2015). The nature of self-regulatory fatigue and “ego depletion”: lessons from physical fatigue. *Pers. Soc. Psychol. Rev.* 20 1–20. 10.1177/1088868315597841 PMC4788579 26228914

[B29] Garrison K. E. Finley A. J. Schmeichel B. J. (2019). Ego depletion reduces attention control: evidence from two high-powered preregistered experiments. *Pers. Soc. Psychol. Bull.* 45 728–739. 10.1177/0146167218796473 30239268

[B30] Gneezy U. List J. A. Wu G. (2006). The uncertainty effect: when a risky prospect is valued less than its worst possible outcome. *Q. J. Econ.* 121 1283–1309. 10.1093/qje/121.4.1283

[B31] Gray J. A. McNaughton N. (2000). *The Neuropsychology of Anxiety: An Enquiry into the Functions of the Septo-Hippocampal System.* Oxford: Oxford University Press.

[B32] Hagger M. S. Chatzisarantis N. L. D. Alberts H. Anggono C. O. Batailler C. Birt A. R. (2016). A multilab preregistered replication of the ego-depletion effect. *Perspect. Psychol. Sci.* 11 546–573. 27474142 10.1177/1745691616652873

[B33] Hagger M. S. Wood C. Stiff C. Chatzisarantis N. L. D. (2010). Ego depletion and the strength model of self-control: a meta-analysis. *Psychol. Bull.* 136 495–525. 10.1037/a0019486 20565167

[B34] Han P. K. J. Klein W. M. P. Arora N. K. (2011). Varieties of uncertainty in health care: a conceptual taxonomy. *Med. Decis. Making* 31 828–838. 10.1177/0272989x10393976 22067431 PMC3146626

[B35] Hirsh J. B. Kang S. K. (2016). Mechanisms of identity conflict: uncertainty, anxiety, and the behavioral inhibition system. *Pers. Soc. Psychol. Rev.* 20 223–244. 10.1177/1088868315589475 26048875

[B36] Inzlicht M. Berkman E. (2015). Six questions for the resource model of control (and some answers). *Soc. Pers. Psychol. Compass* 9 511–524. 10.1111/spc3.12200 28966660 PMC5621751

[B37] Inzlicht M. Gervais W. Berkman E. (2015). *Bias-Correction Techniques Alone Cannot Determine Whether Ego Depletion is Different From Zero: Commentary on Carter, Kofler, Forster, & McCullough, 2015.* Rochester, NY: SSRN.

[B38] Inzlicht M. Schmeichel B. J. (2012). What is ego depletion? Toward a mechanistic revision of the resource model of self-control. *Perspect. Psychol. Sci.* 7 450–463. 10.1177/1745691612454134 26168503

[B39] Inzlicht M. Schmeichel B. J. Macrae C. N. (2014). Why self-control seems (but may not be) limited. *Trends Cogn. Sci.* 18 127–133. 10.1016/j.tics.2013.12.009 24439530

[B40] Jarvis B. G. (2006). *MediaLab (Version 2006)* [Computer Software] New York, NY: Empirisoft Corporation.

[B41] Job V. Dweck C. S. Walton G. M. (2010). Ego depletion – is it all in your head? *Psychol. Sci.* 21 1686–1693. 10.1177/0956797610384745 20876879

[B42] Kahneman D. Tversky A. (1982). Variants of uncertainty. *Cognition* 11 143–157. 10.1016/0010-0277(82)90023-3 7198958

[B43] Kirschbaum C. Pirke K. Hellhammer D. H. (1993). The ‘trier social stress test’ – a tool for investigating psychobiological stress responses in a laboratory setting. *Neuropsychobiology* 28 76–81. 10.1159/000119004 8255414

[B44] Knobloch-Westerwick S. David P. Eastin M. S. Tamborini D. G. (2009). Sports spectators’ suspense: affect and uncertainty in sports entertainment. *J. Commun.* 59 750–767. 10.1111/j.1460-2466.2009.01456.x

[B45] Kurtz J. L. Wilson T. D. Gilbert D. T. (2007). Quantity versus uncertainty: when winning one prize is better than two. *J. Exp. Soc. Psychol.* 43 979–985. 10.1016/j.jesp.2006.10.020

[B46] Kurzban R. Duckworth A. Kable J. W. Myers J. (2013). An opportunity cost model of subjective effort and task performance. *Behav. Brain Sci.* 36 661–679. 10.1017/s0140525x12003196 24304775 PMC3856320

[B47] Lurquin J. H. Michaelson L. E. Barker J. E. Gustavson D. E. von Bastian C. C. Carruth N. P. (2016). No evidence of the ego-depletion effect across task characteristics and individual differences: a pre-registered study. *PLoS One* 11:e0147770. 10.1371/journal.pone.0147770 26863227 PMC4749338

[B48] Milkman K. L. (2012). Unsure what the future will bring? You may overindulge: uncertainty increases the appeal of wants over shoulds. *Organ. Behav. Hum. Decis. Processes* 119 163–176. 10.1016/j.obhdp.2012.07.003

[B49] Milyavskaya M. Inzlicht M. Johnson T. Larson M. J. (2019). Reward sensitivity following boredom and cognitive effort: a high-powered neurophysiological investigation. *Neuropsychologia* 123 159–168. 10.1016/j.neuropsychologia.2018.03.033 29601888

[B50] Muraven M. Shmueli D. Burkley E. (2006). Conserving self-control strength. *J. Pers. Soc. Psychol.* 91 524–537. 10.1037/0022-3514.91.3.524 16938035

[B51] Muraven M. Slessareva E. (2003). Mechanisms of self-control failure: motivation and limited resources. *Pers. Soc. Psychol. Bull.* 29 894–906. 10.1177/0146167203029007008 15018677

[B52] Muraven M. Tice D. M. Baumeister R. F. (1998). Self-control as limited resource: regulatory depletion patterns. *J. Pers. Soc. Psychol.* 74 774–789. 10.1037/0022-3514.74.3.774 9523419

[B53] Orasanu J. Connolly T. (1993). “The reinvention of decision-making,” in *Decision-Making in Action: Models and Methods*, eds Klein G. A. Orasanu J. Calderwood R. Zsambok C. (Norwood, NJ: Ablex), 3–20. 10.1007/978-1-85233-864-0_1

[B54] Park S. H. Glaser J. Kowles E. D. (2008). Implicit motivation to control prejudice moderates the effect of cognitive depletion on unintended discrimination. *Soc. Cogn.* 26 401–419. 10.1521/soco.2008.26.4.401

[B55] Pocheptsova A. Amir O. Dhar R. Baumeister R. F. (2009). Deciding without resources: resource depletion and choice in context. *J. Mark. Res.* 46 344–355. 10.1509/jmkr.46.3.344 11670861

[B56] Pohl R. F. Erdfelder E. Hilbig B. E. Liebke L. Stahlberg D. (2013). Effort reduction after self-control depletion: the role of cognitive resources in use of simple heuristics. *J. Cogn. Psychol.* 25 267–276. 10.1080/20445911.2012.758101

[B57] Polasky S. Carpenter S. R. Folke C. Keeler B. (2011). Decision-making under great uncertainty: environmental management in an era of global change. *Trends Ecol. Evol.* 26 398–404. 10.1016/j.tree.2011.04.007 21616553

[B58] Posen B. R. (2016). Foreword: military doctrine and the management of uncertainty. *J. Strateg. Stud.* 39 159–173. 10.1080/01402390.2015.1115042

[B59] Preacher K. J. Hayes A. (2004). SPSS and SAS procedures for estimating indirect effects in simple mediation models. *Behav. Res. Methods Instrum. Comput.* 36 717–731. 10.3758/bf03206553 15641418

[B60] Preston S. D. Buchanan T. W. Stansfield R. B. Bechara A. (2007). Effects of anticipatory stress on decision making in a gambling task. *Behav. Neurosci.* 121 257–263. 10.1037/0735-7044.121.2.257 17469915

[B61] Redshaw J. Suddendorf T. (2016). Children’s and apes’ preparatory responses to two mutually exclusive possibilities. *Curr. Biol.* 26 1758–1762. 10.1016/j.cub.2016.04.062 27345164

[B62] Rosenthal R. Rubin D. B. (1986). Meta-analytic procedures for combining studies with multiple effects. *Psychol. Bull.* 99 400–406. 10.1037/0033-2909.99.3.400

[B63] Schmeichel B. Vohs K. (2009). Self-affirmation and self-control: affirming core values counteracts ego depletion. *J. Pers. Soc. Psychol.* 96 770–782. 10.1037/a0014635 19309201

[B64] Shafir E. (1994). Uncertainty and the difficulty of thinking through disjunctions. *Cognition* 50 403–430. 10.1016/0010-0277(94)90038-8 8039371

[B65] Shenhav A. Musslick S. Lieder F. Kool W. Griffiths T. L. Cohen J. D. (2017). Toward a rational and mechanistic account of mental effort. *Annu. Rev. Neurosci.* 40 99–124. 10.1146/annurev-neuro-072116-031526 28375769

[B66] Simonsohn U. (2009). Evidence from risky prospects valued below their worst outcome. *Psychol. Sci.* 20 686–692. 10.1111/j.1467-9280.2009.02349.x 19422629

[B67] Starcke K. Wolf O. T. Markowitsch H. J. Brand M. (2008). Anticipatory stress influences decision making under explicit risk conditions. *Behav. Neurosci.* 122 1352–1360. 10.1037/a0013281 19045954

[B68] Stockhammer E. Grafl L. (2010). Financial uncertainty and business investment. *Rev. Polit. Econ.* 22 551–568. 10.1080/09538259.2010.510317

[B69] Thanh B. N. Strobel J. Lee G. (2018). A new measure of real estate uncertainty shocks. *Real Estate Econ.* 48 744–771. 10.1111/1540-6229.12270

[B70] Tice D. M. Baumeister R. F. Shmueli D. Muraven M. (2007). Restoring the self: positive affect helps improve self-regulation following ego depletion. *J. Exp. Soc. Psychol.* 43 379–384. 10.1016/j.jesp.2006.05.007

[B71] Vohs K. D. Baumeister R. F. Schmeichel B. J. (2012). Motivation, personal beliefs, and limited resources all contribute to self-control. *J. Exp. Soc. Psychol.* 48 943–947. 10.1016/j.jesp.2012.03.002

[B72] Vohs K. D. Baumeister R. F. Schmeichel B. J. Twenge J. M. Nelson N. M. Tice D. M. (2014). Making choices impairs subsequent self-control: a limited-resource account of decision making, self-regulation, and active initiative. *Motiv. Sci.* 1 19–42. 10.1037/2333-8113.1.S.19 18444745

[B73] Wiggins S. Whyte P. Huggins M. Adam S. Theilmann J. Bloch M. (1992). The psychological consequences of predictive testing for Huntington’s disease. *N. Engl. J. Med.* 327 1401–1405. 1406858 10.1056/NEJM199211123272001

[B74] Wilson T. D. Centerbar D. B. Kermer D. A. Gilbert D. T. (2005). The pleasures of uncertainty: prolonging positive moods in ways people do not anticipate. *J. Pers. Soc. Psychol.* 88 5–21. 10.1037/0022-3514.88.1.5 15631571

[B75] Xiao S. Dang J. Mao L. Liljedahl S. (2014). When more depletion offsets the ego depletion effect. *Soc. Psychol.* 45 421–425. 10.1027/1864-9335/a000197

