# Supplementary material for: What You Don’t Know Can Hurt You: Uncertainty Impairs Executive Function
Source: Front Psychol. 2020 Oct 6;11:576001. doi: 10.3389/fpsyg.2020.576001 (PMC7573282; doi:10.3389/fpsyg.2020.576001)
Supplement: Supplementary file 1 [file Data_Sheet_1.PDF]

Supplemental Materials for

What you don’t know can hurt you: Uncertainty impairs self-regulation

List of tested mediators and moderators not reported in manuscript

Measures and manipulations

Note: Scales labeled in this document were not labeled for study administration.

Tested Mediators and Moderators

These mediators and moderators were measured in the studies reported in the paper. The results were either not significant or significant in relatively uninteresting ways (e.g. people who know they will have to give a speech plan more to give a speech than people who know they won't have to give a speech). To keep the manuscript succinct, we have left them out of the manuscript, but are providing the analyses here for interested readers. All materials are also included on subsequent pages in the supplemental materials.

Moderators

Neuroticism (Study 1) 2

Intolerance of Uncertainty (Study 1) 3

Need for Closure (Study 3) 3

Thought Control (Study 2) 3

Prevention Focus (Study 2) 4

Promotion Focus (Study 2) 4

Social Anxiety (Study 1) 6

Mediators

Construal Level (Study 1) 6

Time Orientation (Study 1) 6

Thought Intrusion (Study 2) 6

Thinking About the Speech (Study 2) 7

Planning to Give and Rate Speeches (Study 2) 7

Motivation (Study 2)..8

Effort (Study 2)..8

Sense of Control (Study 2)...8

Mood (Studies 2 & 3) 9

Stress, Anxiety, and Worry (Study 1)...9

Moderators

Neuroticism (Study 1)

There was no significant interaction between condition and neuroticism on number of errors on Operation, $F(2, 40) = .55$, $p = .58$, $\eta^2 = .03$, 90% CI[.00, 5.55], or time to complete the task, $F(2, 40) = .004$, $p = .996$, $\eta^2 = .00$, 90% CI[.00, .17]. The effect of condition on errors remained significant in the model including the interaction with neuroticism, $F(2, 40) = 4.18$, $p = .02$, $\eta^2 = .17$, 90% CI[.63, 19.94].

Intolerance of Uncertainty (Study 1)

There was no significant interaction between condition and intolerance of uncertainty on number of errors, $F(2, 40) = 1.24$, $p = .30$, $\eta^2 = .06$, 90% CI[.00, 9.00], or time, $F(2, 40) = .39$, $p = .68$, $\eta^2 = .02$, 90% CI[.00, 4.50]. The effect of the condition on the number of errors remained significant in the model including the interaction with intolerance of uncertainty, $F(2, 40) = 4.01$, $p = .03$, $\eta^2 = .17$, 90% CI[.51, 19.36].

Individual Differences in Thought Control (Study 2)

There was no significant interaction between condition and thought control on number of problems attempted, $F(2, 86) = 2.86$, $p = .06$, $\eta^2 = .06$, 90% CI[.00, 15.13], or solved, $F(2, 86) = 3.11$, $p = .05$, $\eta^2 = .07$, 90% CI[.005, 16.02]. Because the p-values for the interactions were less than .10, we probed the interactions

For participants who reported being high in thought control, there was no significant difference between participants in the uncertain and speech conditions in anagrams attempted, $F(1, 86) = .94$, $p = .34$, $\eta^2 = .01$, 95% CI[.00, 8.59], or anagrams solved, $F(1, 86) = .01$, $p = .91$, $\eta^2 < .001$, 95% CI[00, .43]. Among participants high in thought control, participants attempted, $F(1, 86) = 8.84$, $p = .004$, $\eta^2 = .09$, 95% CI[.91, 24.75], and solved, $F(1, 86) = 8.50$, $p = .005$, $\eta^2 = .09$, 95% CI[.81, 24.16], significantly fewer anagrams in the uncertain condition than in the no speech condition.

Among participants low in thought control, there was no significant difference between participants in the uncertain and speech conditions in anagrams attempted, $F(1, 86) = 3.03$, $p = .09$, $\eta^2 = .03$, 95% CI[.00, 13.79] or solved, $F(1, 86) = 1.50$, $p = .22$, $\eta^2 = .02$, 95% CI[.00, 10.17]. There was also no significant difference between participants in the uncertain and no speech conditions in anagrams attempted, $F(1, 86) = .12$, $p = .74$, $\eta^2 = .001$, 95% CI[.00, 4.95], or solved, $F(1, 86) = .14$, $p = .71$, $\eta^2 = .002$, 95% CI[.00, 5.14].

Prevention Focus (Study 2)

There was no significant interaction between condition and prevention focus on the number of anagrams attempted, $F(2, 86) = 2.12$, $p = .13$, $\eta^2 = .05$, 90% CI[.00, 12.47], or solved, $F(2, 86) = 1.35$, $p = .27$, $\eta^2 = .03$, 90% CI[.00, 9.42].

Promotion Focus (Study 2)

There was no significant interaction between condition and promotion focus on the number of anagrams attempted, $F(2, 86) = .57$, $p = .57$, $\eta^2 = .01$, 90% CI[.00, 5.67], or solved, $F(2, 86) = .47$, $p = .63$, $\eta^2 = .01$, 90% CI[.00, .37].

Social Anxiety (Study 1)

There was no significant interaction between condition and social anxiety on the number of errors participants made on Operation, $F(2, 40) = .19$, $p = .19$, $\eta^2 = .08$, 90% CI[.00, 2.73], or the time they spent on the game, $F(2, 40) = .01$, $p = .99$, $\eta^2 = .001$, 90% CI[.00, .43]. The effect of condition on the number of errors remained significant in the model including the interaction with social anxiety, $F(2, 40) = 4.44$, $p = .02$, $\eta^2 = .18$, 90% CI[.82, 20.81].

Mediators

Construal Level (Study 1)

There were no differences among the conditions on construal level, $F(2, 43) = .25$, $p = .78$, $\eta^2 = .01$, 95% CI[.00, .06]

Time Orientation (Study 1)

There were no differences among conditions on time orientation, $F(43) = .60$, $p = .55$, $\eta^2 = .03$, 95% CI[.00, .11].

Thought Intrusion (Study 2)

There was a significant difference among conditions on thought intrusion, $F(2, 86) = 4.92$, $p = .01$, $\eta^2 = .10$, 90% CI[.02, .20]. Participants in the speech condition reported significantly more intrusive thoughts ($M = 4.80$, $SD = 2.48$) than participants in the uncertain condition ($M = 3.41$, $SD = 2.05$), $t(86) = -2.36$, $p = .02$, $d = -.64$, 95% CI[-2.55, -.22], and no speech condition ($M = 2.87$, $SD = 2.10$), $t(86) = 3.08$, $p = .003$, $d = .89$, 95% CI[.68, 3.18]. There were no significant differences between the uncertain condition and the no speech condition on intrusive thoughts, $t(86) = 1.02$, $p = .31$, $d = .25$, 95% CI[-.52, 1.61].

Thinking About the Speech (Study 2)

There was a significant difference among conditions on thinking about the speech, $F(2, 86) = 3.78$, $p = .03$, $\eta^2 = .08$, 90% CI[.01, .17]. Participants in the speech condition reported thinking significantly more about the speech ($M = 3.43$**,** $SD = 2.29$) than participants in the uncertain condition ($M = 2.23$, $SD = 1.95$), $t(86) = -2.32$, $p = .02$, , $d = -.63$, 95% CI[-2.23, -.18] and no speech condition ($M = 2.00$, $SD = 1.52$), $t(86) = 2.58$, $p = .01$, $d = .74$, 95% CI[.33, 2.53]. There were no significant differences between the uncertain and no speech conditions on thinking about the speech, $t(86) = .48$, $p = .64$, $d = .12$, 95% CI[-.71, 1.16].

Planning to Give and Rate Speeches (Study 2)

There was a significant difference between conditions on planning for giving the speech, $F(2, 86) = 4.82$, $p = .01$, $\eta^2 = .10$, 90% CI[.01, .20]. Participants in the speech condition also reported planning significantly more for the speech ($M = 3.00$, $SD = 2.47$) than participants in the uncertain condition ($M = 1.87$, $SD = 1.59$), $t(86) = -2.29$, $p = .02$, $d = -.62$, 95% CI[-2.10, -.15] and no speech condition ($M = 1.39$, $SD = 1.55$), $t(86) = 3.06$, $p = .003$, $d = .88$, 95% CI[.56,

2.65]. There were no significant differences between the uncertain and no speech conditions on planning for the speech, $t(86) = 1.07$, $p = .29$, $d = .25$, 95% CI[-.41, 1.37]. There were no significant differences among conditions on the amount that participants indicated that they were planning to rate the speech, $F(2, 86) = .78$, $p = .46$, $\eta^2 = .02$, 90% CI[.00, .07].

Motivation (Study 2)

There were no significant differences among conditions on motivation, $F(2, 86) = .25$, $p = .78$, $\eta^2 = .01$, 90% CI[.00, .04].

Effort (Study 2)

There were no significant differences among conditions on effort, $F(2, 86) = .44$, $p = .65$, $\eta^2 = .01$, 90% CI[.00, .05].

Sense of Control (Study 2)

There were no significant differences between conditions on the amount of control participants felt they had, $F(2, 86) = .50$, $p = .61$, $\eta^2 = .01$, 90% CI[.00, .06].

Mood (Study 2)

There were no significant differences among conditions on mood, $F(2, 86) = 1.03$, $p = .36$, $\eta^2 = .02$, 90% CI[.00, .08].

Stress, Anxiety and Worry (Study 2)

There were significant differences among conditions on self-reported feelings of stress, $F(2, 86) = 5.19$, $p = .007$, $\eta^2 = .11$, 90% CI[.02, .20], anxiety, $F(2, 86) = 6.41$, $p = .003$, $\eta^2 = .13$, 90% CI[.03, .23], and worry, $F(2, 86) = 8.62$, $p < .001$, $\eta^2 = .17$, 90% CI[.05, .27].

Participants in the speech condition ($M = 5.10$, $SD = 2.23$) reported significantly more stress than participants in the uncertain condition ($M = 3.90$, $SD = 1.85$), $t(86) = -2.18$, $p = .03$, $d = - .59$, 95% CI[-2.28, -.11], and no speech condition ($M = 3.21$, $SD = 2.11$), $t(86) = 3.21$, $p = .002$, $d = .93$, 95% CI[.72, 3.05]. Participants in the speech condition also reported feeling significantly more anxious ($M = 5.24$, $SD = 2.17$) than participants in the uncertain condition ($M = 3.58$, $SD = 2.05$), $t(86) = -3.00$, $p = .004$, $d = -.81$, 95% CI[-2.76, -.56], and no speech condition ($M = 3.23$, $SD = 1.98$), $t(86) = 3.38$, $p = .001$, $d = .98$, 95% CI[.83, 3.19]. Participants in the speech condition also reported feeling more worried ($M = 4.67$, $SD = 2.06$) than participants in the uncertain condition ($M = 3.03$, $SD = 1.89$), $t(86) = -3.15$, $p = .002$, $d = -.85$, 95% CI[-2.68, -.61], and no speech condition ($M = 2.39$, $SD = 1.91$), $t(86) = 4.07$, $p < .001$, $d = 1.17$, 95% CI[1.16, 3.39].

There were no significant differences between the uncertain condition and the no speech condition on stress, $t(86) = 1.37$, $p = .17$, $d = .34$, 95% CI[-.31, 1.68], anxiety, $t(86) = .68$, $p = .50$, $d = .17$, 95% CI[-.66, 1.35], or worry, $t(86) = 1.32$, $p = .19$, $d = .33$, 95% CI[-.32, 1.58]. Participants were more stressed, anxious, and worried in the speech condition than in the uncertain or no speech condition. Participants in the uncertain condition did not report feeling significantly more stressed, anxious, or worried than participants in the no speech condition.

Measures and Manipulations Administered for

Study 1

Note: The following scales were administered one item at a time on a computer. The order of the items within each of the three individual difference scales that follow were randomized.

Intolerance of Uncertainty Scale (Buhr & Dugas, 2002)

1	2	3	4	5
Not at all characteristic of me				Entirely characteristic of me

Uncertainty stops me from having a strong opinion.
Being uncertain means that a person is disorganized.
Uncertainty makes life intolerable.
It’s unfair having no guarantees in life.
My mind can’t be relaxed if I don’t know what will happen tomorrow.
Uncertainty makes me uneasy, anxious, or stressed.
Unforeseen events upset me greatly.
IT frustrates me not having all the information I need.
Uncertainty keeps me from living a full life.
One should always look ahead so as to avoid surprises.
A small unforeseen event can spoil everything, even with the best planning.
When it’s time to act, uncertainty paralyses me.
Being uncertain means that I am not first rate.
When I am uncertain, I can’t go forward.
When I am uncertain, I can’t function very well.
Unlike me, others seem to know where they are going with their lives.
Uncertainty makes me vulnerable, unhappy, or sad.
I always want to know what the future has in store for me.
I can’t stand being taken by surprise.
The smallest doubt can stop me from acting.
I should be able to organize everything in advance.
Being uncertain means that I lack confidence.
I think it’s unfair that other people seem to be sure about their future.
Uncertainty keeps me from sleeping soundly.
I must get away from all uncertain situations.
The ambiguities in life stress me.
I can’t stand being undecided about my future.

Social Interaction Anxiety Scale (Mattick & Clarke, 1998)

0	1	2	3	4
Not at all characteristic of me	Slightly characteristic of me	Moderately characteristic of me	Very characteristic of me	Extremely characteristic of me

I get nervous if I have to speak with someone in authority (teacher, boss, etc.).
I have difficulty making eye contact with others.
I become tense if I have to talk about myself or my feelings.
I find difficulty mixing comfortably with people I work with.
I find it easy to make friends of my own age.
I tense up if I meet an acquaintance on the street.
When mixing socially, I am uncomfortable.
I feel tense if I am alone with just one person.
I am at ease meeting people at parties, etc.
I have difficulty talking with other people.
I find it easy to think of things to talk about.
I worry about expressing myself in case I appear award.
I find it difficult to disagree with another's point of view.
I have difficulty talking to an attractive person of the opposite sex.
I find myself worrying that I won't know what to say in social situations.
I am nervous mixing with people I don't know well.
I feel I'll say something embarrassing when talking.
When mixing in a group, I find myself worrying I will be ignored.
I am tense mixing in a group.
I am unsure whether to greet someone I know only slightly.

Neuroticism subscale of the International Personality Item Pool (Goldberg, 1999)

1	2	3	4	5
Not at all characteristic of me	Slightly characteristic of me	Moderately characteristic of me	Very characteristic of me	Extremely characteristic of me

Using the scale provided, please rate the extent to which each statement applies to you.

I get angry easily.
I rarely get irritated.
I get upset easily.
I keep my emotions under control.
I change my mood a lot.
I rarely lose my composure.
I am a person whose moods go up and down easily.
I am not easily annoyed.
I get easily agitated.
I can be stirred up easily.
I seldom feel blue.
I am filled with doubts about things.
I feel comfortable with myself.
I feel threatened easily.
I rarely feel depressed.
I worry about things.
I am easily discouraged.
I am not embarrassed easily.
I become overwhelmed by events.
I am afraid of many things.

Note: Uncertainty was manipulated via the experimenter's instructions after the individual difference scales were administered and before the measures that follow.

Construal Level (Fujita, Trope, Liberman, & Levin-Sagi, 2006)

For each item below, you will see two descriptions of the same action. Please choose the statement that best describes the action.

Sweeping the floor
- Moving a broom
- Being clean

Attending a family reunion
- Going to a picnic
- Respecting tradition

Skydiving
- Jumping out of a plane
- Demonstrating one's daringness

Making an expensive purchase
- Swiping a credit card
- Doing something for one's pleasure

Staying home to study
- Reviewing one's notes
- Exerting self-discipline

Recycling
- Bagging paper, glass, and cans
- Caring for the environment

Teaching
- Talking to students
- Having authority

Meeting new people
- Small talk and shaking hands
- Enhancing one's social network

Time orientation self-report (adapted from Twenge, Catanese, & Baumeister, 2003; Kuhlen & Monge, 1968; Gjesme, 1979)

Please rate your agreement with each of the following statements

1	2	3	4	5	6	7	8	9
Strongly disagree		Disagree		Neither agree nor disagree		Agree		Strongly agree

I can only think about the present

I find it difficult to think about the future

I am most concerned about how I feel in the present.

I feel a strong tendency to enjoy myself today and let the future take care of itself.

Note: When I began the study, I also included a measure of working memory (Hoffman et al., 2008). However, after 32 participants, only one participant was able to correctly reproduce the set of numbers, so the measure was cut.

Final Measures

Before we continue on to the speech task, we'd like your opinion on the tasks you've completed so far. Please answer the following questions as honestly as possible.

What do you think the study was measuring?

Did anything unusual happen during your session?

How much did you enjoy playing the game Operation?

1	2	3	4	5
Not at all	A little	Moderately	Very much	Extremely

How much have you enjoyed the study as a whole so far?

1	2	3	4	5
Not at all	A little	Moderately	Very much	Extremely

How friendly was the researcher who administered your study today?

1	2	3	4	5
Not at all	A little	Moderately	Very	Extremely

How competent was the researcher who administered your study today?

1	2	3	4	5
Not at all	A little	Moderately	Very much	Extremely

I am:

Male

Female

Please enter your age:

I would describe my ethnicity as:

Hispanic or Latino

Not Hispanic or Latino

I would describe my race as

American Indian/Alaskan Native

Asian

Native Hawaiian or Other Pacific Islander

Black or African American

White

More than one race

Unknown or not reported

For the experimenter: Please describe anything unusual that happened during this study.

Measures and Manipulations Administered for

Study 2

Note: The following measures were given to participants after the uncertainty manipulation.

Please solve as many of the following anagrams as possible in 5 minutes.

1	ETAWS	____________	26	NITSK	____________
2	HUNCL	____________	27	AKYLF	____________
3	OTAGN	____________	28	ZYUFZ	____________
4	LICHD	____________	29	LESHL	____________
5	SEALF	____________	30	SASCL	____________
6	RUTOC	____________	31	LIEOV	____________
7	GANIL	____________	32	RCMEY	____________
8	ROGOM	____________	33	OIEDV	____________
9	TTRHU	____________	34	EESAT	____________
10	MGIIC	____________	35	RDPIA	____________
11	EIUTQ	____________	36	TYRAP	____________
12	CMIAG	____________	37	RCOOL	____________
13	ESRIN	____________	38	ITNPA	____________
14	BEETR	____________	39	ETESH	____________
15	PLAEP	____________	40	NIJOT	____________
16	EDNOZ	____________	41	NRCOS	____________
17	HOINR	____________	42	RSWAE	____________
18	NTALP	____________	43	COKKN	____________
19	OHBOT	____________	44	KAETN	____________
20	LUGAH	____________	45	OKSOH	____________
21	NUSYN	____________	46	LIKCC	____________
22	KNCHU	____________	47	OMESO	____________
23	RYRBE	____________	48	TASSH	____________
24	KOLAP	____________	49	OAHCS	____________
25	EIHGW	____________	50	RUHYR	____________

White Bear Suppression Inventory (Wegner & Zanakos, 1994)

1	2	3	4	5
Strongly disagree	Disagree	Neutral	Agree	Strongly Agree

This survey is about thoughts. There are no right or wrong answers, so please respond honestly to each of the items below.

_____________ There are things I prefer not to think about.

_____________ Sometimes I wonder why I have the thoughts I do.

_____________ I have thoughts that I cannot stop.

_____________ There are images that come to mind that I cannot erase.

_____________ My thoughts frequently return to one idea.

_____________ I wish I could stop thinking of certain things.

_____________ Sometimes my mind races so fast I wish I could stop it.

_____________ I always try to put problems out of mind.

_____________ There are thoughts that keep jumping into my head.

_____________ There are things that I try not to think about.

_____________ Sometimes I really wish I could stop thinking.

_____________ I often do things to distract myself from my thoughts.

_____________ I have thoughts that I try to avoid.

_____________ There are many thoughts that I have that I don’t tell anyone.

_____________ Sometimes I stay busy just to keep thoughts from intruding on my mind.

Promotion & Prevention Focus (Lockwood, Jordan, & Kunda, 2002)

1	2	3	4	5	6	7	8	9
Not at all true of me								Very true of me

Please rate your agreement with each statement on the scale provided.

1__________ In general, I am focused on preventing negative events in my life.

2__________ I am anxious that I will fall short of my responsibilities and obligations.

3__________ I frequently imagine how I will achieve my hopes and aspirations.

4__________ I often think about the person I am afraid I might become in the future.

5__________ I often think about the person I would ideally like to be in the future.

6__________ I typically focus on the success I hope to achieve in the future.

7__________ I often worry that I will fail to accomplish my academic goals.

8__________ I often think about how I will achieve academic success.

9__________ I often imagine myself experiencing bad things that I fear might happen to me.

10__________I frequently think about how I can prevent failures in my life.

11__________I am more oriented toward preventing losses than I am toward achieving gains.

12__________My major goal in school right now is to achieve my academic ambitions.

13__________My major goal in school right now is to avoid becoming an academic failure.

14__________I see myself as someone who is primarily striving to reach my "ideal self" – to fulfill my hopes, wishes and aspirations.

15__________I see myself as someone who is primarily striving to become the self I "ought" to be – to fulfill my duties, responsibilities, and obligations.

16__________In general, I am focused on achieving positive outcomes in my life.

17__________I often imagine myself experiencing good things that I hope will happen to me

18__________Overall, I am oriented toward achieving success than prevent failure.

Potential Mediators

Thought intrusion (some items adapted from Creamer, Bell, & Failla, 2003)

Please rate your agreement with the following statements

1	2	3	4	5	6	7	8	9
Strongly Disagree		Slightly disagree		Neither agree nor disagree		Slightly agree		Strongly agree

___________ During the anagram task, I was trying to avoid thinking about the speech task.

___________ During the anagram task, I couldn’t stop thinking about the speech task.

___________ During the anagram task, I thought about the speech task when I didn’t mean to.

___________ During the anagram task, I had trouble concentrating.

Please indicate the frequency of your thoughts using the scale below:

1	2	3	4	5	6	7	8	9
Not at all		A little bit of the time		Some of the time		Most of the time		Constantly

___________ During the anagram task, how often were you thinking about the speech task?

___________ During the anagram task, how often were you planning what you would be doing if you gave a speech?

___________ During the anagram task, how often were you planning were you planning what you would be doing if you were rating speeches?

___________ During this study, how often were you thinking about things other than the study?

Please circle your answer to the following questions on the scale provided.

How motivated were you to do the anagram task?

1	2	3	4	5	6	7	8	9
Not at all motivated		A little motivated		Somewhat motivated		Very motivated		Extremely motivated

How much effort did you put into the anagram task?

1	2	3	4	5	6	7	8	9
No effort		A little effort		Some effort		A lot of effort		An extreme amount of effort

How difficult was it to do the anagram task?

1	2	3	4	5	6	7	8	9
Not at all difficult		A little difficult		Somewhat difficult		Very difficult		Extremely difficult

How tired were you before beginning the anagram task?

1	2	3	4	5	6	7	8	9
Not at all tired		A little tired		Somewhat tired		Very tired		Extremely tired

---

How much control did you feel like you had during the study?

1	2	3	4	5	6	7	8	9
No control		A little control		Some control		A lot of control		An extreme amount of control

---

Please rate your agreement with the following statements on the scale provided.
I hate public speaking.

1	2	3	4	5	6	7	8	9
Strongly Disagree		Slightly disagree		Neither agree nor disagree		Slightly agree		Strongly agree

I feel confident giving a speech in front of others.

1	2	3	4	5	6	7	8	9
Strongly Disagree		Slightly disagree		Neither agree nor disagree		Slightly agree		Strongly agree

---

Think about how you felt during the anagram task.
How uncertain did you feel during the anagram task?

1	2	3	4	5	6	7	8	9
Not at all uncertain		A little uncertain		Somewhat uncertain		Very uncertain		Extremely uncertain

What were you told that you would be doing later in the study? (circle one)

1	2	3
Giving a speech	Rating a speech	Uncertain

Earlier in the study, I was uncertain about whether or not I'd be giving a speech.

1	2	3	4	5	6	7	8	9
Strongly disagree		Somewhat disagree		Neither agree nor disagree		Somewhat agree		Strongly agree

Think about how you felt during the anagram task.
How POSITIVE or NEGATIVE was your mood during the anagram task?

1	2	3	4	5	6	7	8	9
Extremely negative		Somewhat negative		Neutral		Somewhat positive		Extremely positive

Think about how you felt during the anagram task.
How stressed did you feel during the anagram task?

1	2	3	4	5	6	7	8	9
Not at all stressed		A little stressed		Somewhat stressed		Very stressed		Extremely stressed

Think about how you felt during the anagram task.
How anxious did you feel during the anagram task?

1	2	3	4	5	6	7	8	9
Not at all anxious		A little anxious		Somewhat anxious		Very anxious		Extremely anxious

Think about how you felt during the anagram task.
How worried did you feel during the anagram task?

1	2	3	4	5	6	7	8	9
Not at all worried		A little worried		Somewhat worried		Very worried		Extremely worried

Final Materials

Please describe anything unusual that happened in your session.

How friendly was the researcher who administered your study today?

1	2	3	4	5	6	7	8	9
Not at all		A little		Somewhat		Very		Extremely

How competent was the researcher who administered your study today?

1	2	3	4	5	6	7	8	9
Not at all		A little		Somewhat		Very		Extremely

When you were doing the anagram task, to what extent did you believe there would be a speech task? (circle one)

I knew there would be no speech task

I doubted that there would be a speech task

I wondered if there really was a speech task

I was pretty sure there would be a speech task

I was confident there would be a speech task

If you didn't believe that there would be a speech task, please tell us why. Is there anything that could be done better in this study to make it more believable?

Thank you for your help!

Please provide the following information about yourself:

Sex ______________________________

Age _____________

I would describe my ethnicity as: (circle one)

Hispanic or Latino   Not Hispanic or Latino

I would describe my race as: (circle one)

American Indian/Alaska Native

Asian

Native Hawaiian or Other Pacific Islander

Black or African American

White

More than one race

Unknown or not reported

Measures and Manipulations Administered for

Study 3

Note: The need for closure scale was administered on the computer.
Need for Closure Scale

Using the scale below, please rate the extent to which you agree with each statement.

Strongly Disagree	Disagree	Slightly Disagree	Slightly Agree	Agree	Strongly Disagree
1	2	3	4	5	6

I think that having clear rules and order at work is essential for success.
Even after I've made up my mind about something, I am always eager to consider a different opinion.
I don't like situations that are uncertain.
I dislike questions which could be answered in many different ways.
I *like* to have friends who are unpredictable.
I find that a well ordered life with regular hours suits my temperament.
I enjoy the uncertainty of going into a new situation without knowing what might happen.
When dining out, I like to go to places where I have been before so that I know what to expect.
I feel uncomfortable when I don't understand the reason why an event occurred in my life.
I feel irritated when one person disagrees with what everyone else in a group believes.
I hate to change my plans at the last minute.
I would describe myself as indecisive.
When I go shopping, I have difficulty deciding exactly what it is that I want.
When faced with a problem I usually see the one best solution very quickly.
When I am confused about an important issue, I feel very upset.
I tend to put off making important decisions until the last possible moment.
I usually make important decisions quickly and confidently.
I have never been late for an appointment or work.
I think it is fun to change my plans at the last moment.
My personal space is usually messy and disorganized.
In most social conflicts, I can easily see which side is right and which is wrong.
I have never known someone I did not like.
I tend to struggle with most decisions.
I believe that orderliness and organization are among the most important characteristics of a good student.
When considering most conflict situations, 1 can usually see how both sides could be right.
I don't like to be with people who are capable of unexpected actions.
I prefer to socialize with familiar friends because I know what to expect from them.
I think that I would learn *best* in a class that *lacks* clearly stated objectives and requirements.
When thinking about a problem, I consider as many different opinions on the issue as possible.
I don’t like to go into a situation without knowing what I can expect from it.
I like to know what people are thinking all the time.
I dislike it when a person's statement could mean many different things.
It's annoying to listen to someone who cannot seem to make up his or her mind.
I find that establishing a consistent routine enables me to enjoy life more.
I enjoy having a clear and structured mode of life.

I *prefer* interacting with people whose opinions are very different from my own.
I like to have a place for everything and everything in its place.
I feel uncomfortable when someone's meaning or intention is unclear to me.
I believe that one should never engage in leisure activities.
When trying to solve a problem I often see so many possible options that it's confusing.
I always see many possible solutions to problems I face.
I'd rather know bad news than stay in a state of uncertainty.
I feel that there is no such thing as an honest mistake.
I do not usually consult many different opinions before forming my own view.
I dislike unpredictable situations.
I have never hurt another person’s feelings.
I dislike the routine aspects of my work (studies).

After completing that scale, the manipulation was administered on the computer according to the description in the methods section of the paper. The boxes were large on the screen. They are smaller in this packet to conserve space.

Uncertain Condition

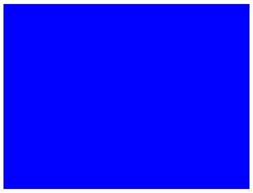

Please complete the equation associated with the color you just saw:
Blue: 3 x 5 =
Green: 6 x 7 =
Yellow: 7 x 9 =
Red: 3 x 8 =

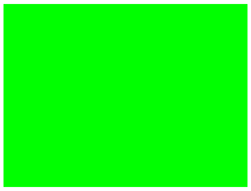

Please complete the equation associated with the color you just saw:
Blue: 10 x 4 =
Green: 7 x 2 =
Yellow: 9 x 6 =
Red: 4 x 7 =

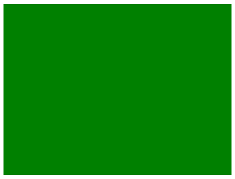

Please complete the equation associated with the color you just saw:
Blue: 3 x 2 =
Green: 4 x 9 =
Yellow: 5 x 11 =
Red: 8 x 8 =

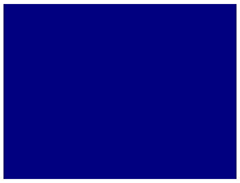

Please complete the equation associated with the color you just saw:
Blue: 5 x 4 =
Green: 12 x 7 =
Yellow: 9 x 3 =
Red: 11 x 6 =

Please complete the equation associated with the color you just saw:
Blue: 2 x 6 =
Green: 12 x 3 =
Yellow: 10 x 7 =
Red: 9 x 9 =

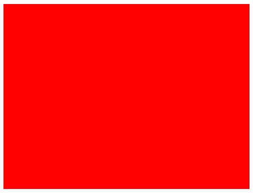

Please complete the equation associated with the color you just saw:
Blue: 11 x 8 =
Green: 6 x 5 =
Yellow: 4 x 3 =
Red: 7 x 6 =

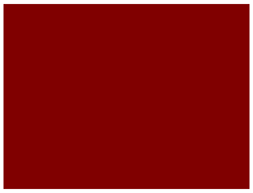

Please complete the equation associated with the color you just saw:
Blue: 2 x 2 =
Green: 5 x 6 =
Yellow: 12 x 9 =
Red: 7 x 4 =

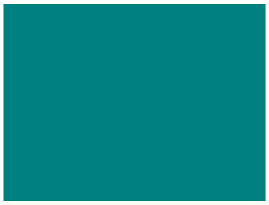

Please complete the equation associated with the color you just saw:
Blue: 9 x 11 =
Green: 8 x 3 =
Yellow: 5 x 5 =
Red: 4 x 7 =

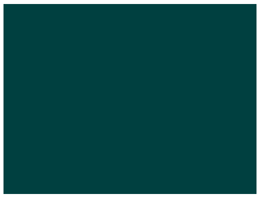

Please complete the equation associated with the color you just saw:
Blue: 11 x 3 =
Green: 6 x 2 =
Yellow: 5 x 9 =
Red: 8 x 4 =

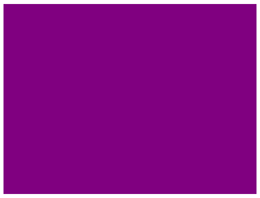

Please complete the equation associated with the color you just saw:
Blue: 5 x 3 =
Green: 6 x 6 =
Yellow: 8 x 9 =
Red: 10 x 7 =

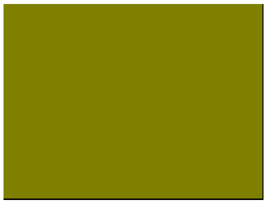

Please complete the equation associated with the color you just saw:
Blue: 6 x 3 =
Green: 10 x 5 =
Yellow: 9 x 6 =
Red: 5 x 8 =

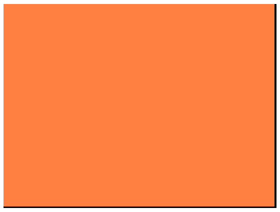

Please complete the equation associated with the color you just saw:
Blue: 11 x 4 =
Green: 7 x 5 =
Yellow: 4 x 8 =
Red: 7 x 7 =

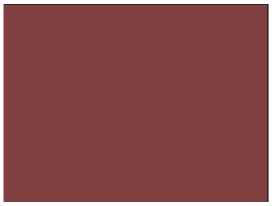

Please complete the equation associated with the color you just saw:
Blue: 8 x 5 =
Green: 5 x 7 =
Yellow: 10 x 8 =
Red: 6 x 4 =

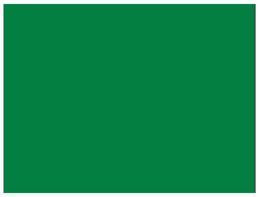

Please complete the equation associated with the color you just saw:
Blue: 8 x 6 =
Green: 4 x 4 =
Yellow: 12 x 5 =
Red: 4 x 10 =

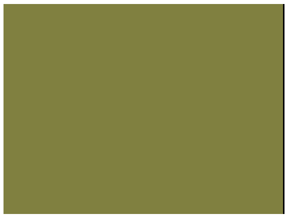

Please complete the equation associated with the color you just saw:
Blue: 8 x 7 =
Green: 2 x 9 =
Yellow: 6 x 11 =
Red: 3 x 7 =

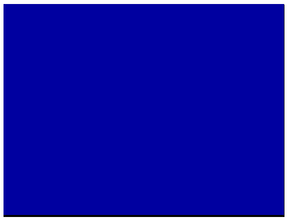

Please complete the equation associated with the color you just saw:
Blue: 8 x 2 =
Green: 4 x 12 =
Yellow: 10 x 9 =
Red: 2 x 8 =

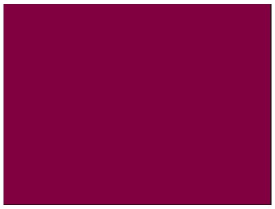

Please complete the equation associated with the color you just saw:
Blue: 10 x 2 =
Green: 3 x 3 =
Yellow: 7 x 8 =
Red: 10 x 3 =

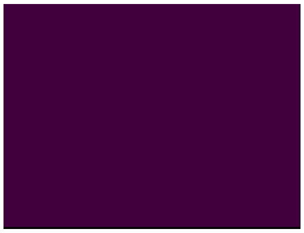

Please complete the equation associated with the color you just saw:
Blue: 4 x 2 =
Green: 9 x 7 =
Yellow: 12 x 6 =
Red: 3 x 9 =

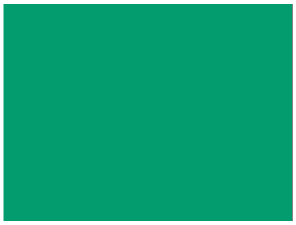

Please complete the equation associated with the color you just saw:
Blue: 9 x 2 =
Green: 10 x 6 =
Yellow: 2 x 4 =
Red: 9 x 5 =

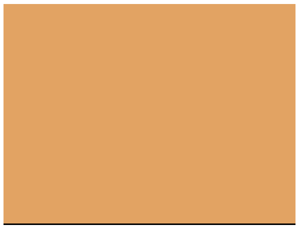

Please complete the equation associated with the color you just saw:
Blue: 2 x 12 =
Green: 7 x 3 =
Yellow: 8 x 10 =
Red: 11 x 5 =

Control Condition

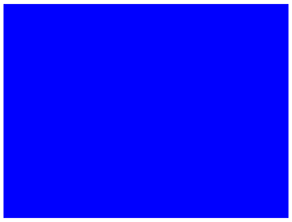

Please complete the equation associated with the color you just saw:
Blue: 3 x 5 =
Green: 6 x 7 =
Yellow: 7 x 9 =
Red: 3 x 8 =

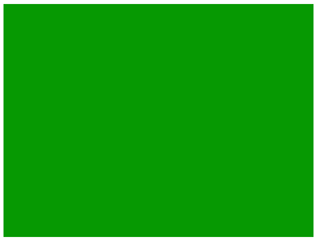

Please complete the equation associated with the color you just saw:
Blue: 10 x 4 =
Green: 7 x 2 =
Yellow: 9 x 6 =
Red: 4 x 7 =

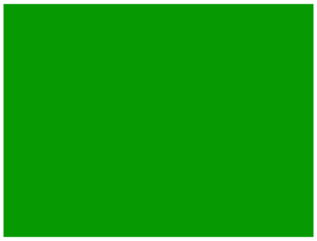

Please complete the equation associated with the color you just saw:
Blue: 3 x 2 =
Green: 4 x 9 =
Yellow: 5 x 11 =
Red: 8 x 8 =

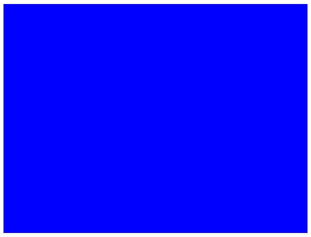

Please complete the equation associated with the color you just saw:
Blue: 5 x 4 =
Green: 12 x 7 =
Yellow: 9 x 3 =
Red: 11 x 6 =

Please complete the equation associated with the color you just saw:
Blue: 2 x 6 =
Green: 12 x 3 =
Yellow: 10 x 7 =
Red: 9 x 9 =

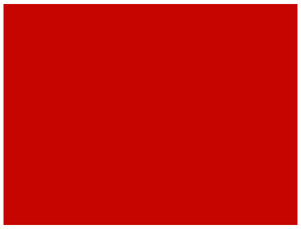

Please complete the equation associated with the color you just saw:
Blue: 11 x 8 =
Green: 6 x 5 =
Yellow: 4 x 3 =
Red: 7 x 6 =

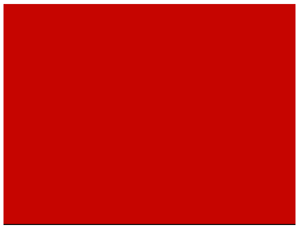

Please complete the equation associated with the color you just saw:
Blue: 2 x 2 =
Green: 5 x 6 =
Yellow: 12 x 9 =
Red: 7 x 4 =

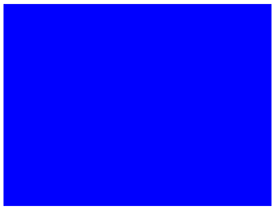

Please complete the equation associated with the color you just saw:
Blue: 9 x 11 =
Green: 8 x 3 =
Yellow: 5 x 5 =
Red: 4 x 7 =

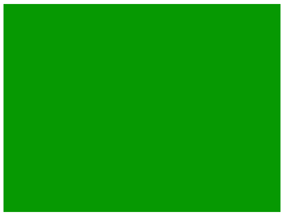

Please complete the equation associated with the color you just saw:
Blue: 11 x 3 =
Green: 6 x 2 =
Yellow: 5 x 9 =
Red: 8 x 4 =

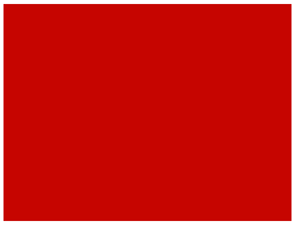

Please complete the equation associated with the color you just saw:
Blue: 5 x 3 =
Green: 6 x 6 =
Yellow: 8 x 9 =
Red: 10 x 7 =

Please complete the equation associated with the color you just saw:
Blue: 6 x 3 =
Green: 10 x 5 =
Yellow: 9 x 6 =
Red: 5 x 8 =

Please complete the equation associated with the color you just saw:
Blue: 11 x 4 =
Green: 7 x 5 =
Yellow: 4 x 8 =
Red: 7 x 7 =

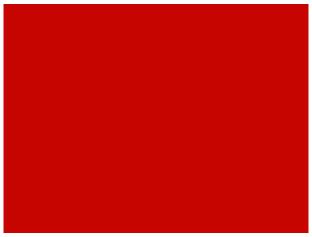

Please complete the equation associated with the color you just saw:
Blue: 8 x 5 =
Green: 5 x 7 =
Yellow: 10 x 8 =
Red: 6 x 4 =

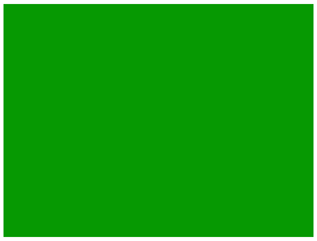

Please complete the equation associated with the color you just saw:
Blue: 8 x 6 =
Green: 4 x 4 =
Yellow: 12 x 5 =
Red: 4 x 10 =

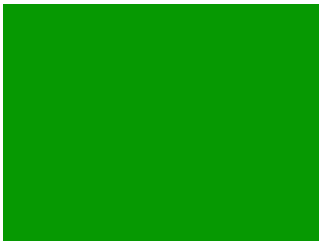

Please complete the equation associated with the color you just saw:
Blue: 8 x 7 =
Green: 2 x 9 =
Yellow: 6 x 11 =
Red: 3 x 7 =

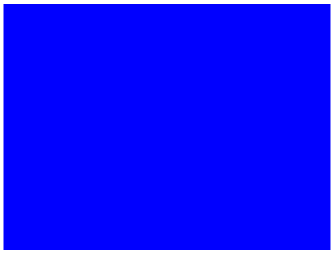

Please complete the equation associated with the color you just saw:
Blue: 8 x 2 =
Green: 4 x 12 =
Yellow: 10 x 9 =
Red: 2 x 8 =

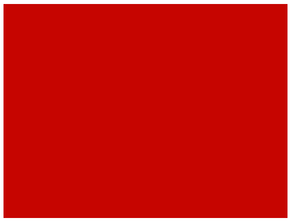

Please complete the equation associated with the color you just saw:
Blue: 10 x 2 =
Green: 3 x 3 =
Yellow: 7 x 8 =
Red: 10 x 3 =

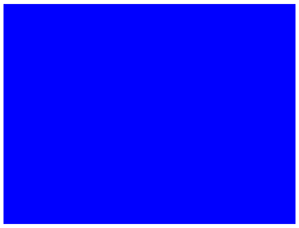

Please complete the equation associated with the color you just saw:
Blue: 4 x 2 =
Green: 9 x 7 =
Yellow: 12 x 6 =
Red: 3 x 9 =

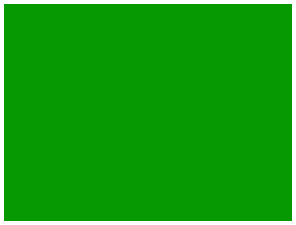

Please complete the equation associated with the color you just saw:
Blue: 9 x 2 =
Green: 10 x 6 =
Yellow: 2 x 4 =
Red: 9 x 5 =

Please complete the equation associated with the color you just saw:
Blue: 2 x 12 =
Green: 7 x 3 =
Yellow: 8 x 10 =
Red: 11 x 5 =

Note: The items of the PANAS and the Felt Uncertainty Scale were randomized and administered on the computer.

PANAS (Watson, Clark, & Tellegen, 1988)

1	2	3	4	5
Not at all	Slightly	Moderately	Quite a bit	Extremely

Distressed
Excited
Upset
Strong
Guilty
Scared
Hostile
Enthusiastic
Proud
Irritable
Alert
Ashamed
Inspired
Nervous
Determined
Attentive
Jittery
Active
Afraid

Felt Uncertainty (McGregor et al., 2001)

1	2	3	4	5
Not at all	Slightly	Moderately	Quite a bit	Extremely

Mixed
Uneasy
Torn
Bothered
Preoccupied
Confused
Unsure of self or goals
Contradictory
Distractable
Unclear
Of two minds
Muddled
Restless
Confused about identity
Jumbled
Uncomfortable
Conflicted
Indecisive
Chaotic
